# The Implication of Substance P in the Development of Tendinopathy: A Case Control Study

**DOI:** 10.3390/ijms18061241

**Published:** 2017-06-09

**Authors:** Soo-Hong Han, Wonchul Choi, Jiye Song, Jaehee Kim, Seungyong Lee, Youngrak Choi, Seong-Eun Byun, Taekeun Ahn, Heejung Ahn, Catherine Ding, Lloyd Baik, Spencer Ward, Kang Ting, Soonchul Lee

**Affiliations:** 1 Department of Orthopedic Surgery, CHA Bundang Medical Center, CHA University, Gyeonggi-do 13496, Korea; hsoohong@hanmail.net (S.-H.H.); wcosdoc@gmail.com (W.C.); eternal-pray@hanmail.net (J.K.); yongiii@naver.com (S.L.); jeanguy@chamc.co.kr (Y.C.); sonofos@cha.ac.kr (S.-E.B.); ajh329@gmail.com (T.A.); 2 Institute for Clinical Research, Department of Orthopedic Surgery, CHA Bundang Medical Center, CHA University, Gyeonggi-do 13496, Korea; songjy2001@naver.com; 3 Department of Pathology, CHA Bundang Medical Center, CHA University School of Medicine, Gyeonggi-do 13496, Korea; hjahn@cha.ac.kr; 4 Division of Growth and Development and Section of Orthodontics, School of Dentistry, University of California, Los Angeles, CA 90095, USA; catherineding@ucla.edu (C.D.); lloydbaik@ucla.edu (L.B.); spencersward3109@gmail.com (S.W.); kting@dentistry.ucla.edu (K.T.)

**Keywords:** tendinopathy, pathogenesis, substance P, type 3 collagen

## Abstract

It was reported that substance P had beneficial effects in the healing of acute tendon injury. However, the relationship between substance P and degenerative tendinopathy development remains unclear. The purpose of this study was to determine the role of substance P in the pathogenesis of tendinopathy. Healthy and tendinopathy tendon were harvested from human and tenocytes were cultured individually. The expression levels of genes associated with tendinopathy were compared. Next, substance P was exogenously administered to the healthy tenocyte and the effect was evaluated. The results showed that tendinopathy tenocytes had higher levels of *COL3A1*, *MMP1*, *COX2*, *SCX*, *ACTA2*, and substance P gene expression compared to healthy tenocytes. Next, substance P treatment on the healthy tenocyte displayed similar changes to that of the tendinopathy tenocytes. These differences between the two groups were also determined by Western blot. Additionally, cells with substance P had the tendinopathy change morphologically although cellular proliferation was significantly higher compared to that of the control group. In conclusion, substance P enhanced cellular proliferation, but concomitantly increased immature collagen (type 3 collagen). Substance P plays a crucial role in tendinopathy development and could be a future therapeutic target for treatment.

## 1. Introduction

Tendinopathy is a chronic degenerative musculoskeletal condition that affects various parts of the body including the Achilles, patellar, rotator cuff, and forearm extensor tendons. This condition is a concern in the developed world since increased involvement in recreational sports is correlated with the rise of tendinopathy incidence [1]. Runners, for example, have a 30% risk of exhibiting Achilles tendinopathy with an annual incidence of about 7–9% [2].

While it is known that tendinopathy is mainly caused by the accumulation of small-scale injuries that do not heal properly, the exact pathogenesis is poorly understood. Theories suggest that the causes are likely to be multifactorial, which may include ischemia, oxygen-free radical injuries, imbalance between vasoconstrictor and vasodilator innervations, micro-tears, and degenerative changes [3,4]. Recently, researchers have reported that neuronal regulation by various neuro-mediators such as substance P (SP) and calcitonin gene-related peptide (CGRP) play an essential role in tendon homeostasis and the disruption of this regulation contributes to the development of neurogenic inflammation and tendinopathy [5,6,7,8]. Neurogenic inflammation arises from the local release of neuro-mediators and can explain why patients with tendinopathy experience symptoms of inflammation such as pain and edema despite the lack of infiltration of inflammatory cells such as macrophages and lymphocytes. Under healthy conditions, neuro-mediators are found only in the surrounding structures of intact tendons such as the paratenon, endotenon, and epitenon. The tendon proper, in contrast, is practically devoid of neuro-mediators. However, in tendinopathy, there is extensive nerve ingrowth and up-regulation of neuro-mediators into the tendon proper [9,10,11,12,13,14]. It has also been shown that tenocytes have the ability to produce neuro-mediators on their own in tendinopathy [15]. In acute tendon injury or rupture, SP is known to be involved in tissue healing through multiple functions such as inflammatory response, pain transmission, increased vascular permeability and blood flow, and cellular proliferation [6,16,17,18]. However, the actual role of SP among the various neuro-mediators in the pathogenesis of degenerative tendinopathy (e.g., chronic tendon injury) is still being debated.

In this experiment, we hypothesized that although SP can play a role in cellular proliferation, it also has the potential to induce a healthy tenocyte into a tendinopathy tenocyte. To test this hypothesis, we first harvested and cultured healthy and tendinopathy tenocytes, then compared the expression levels of several genes and proteins known to be associated with the pathogenesis of tendinopathy. SP was then exogenously administered to the cultured healthy tenocyte and its effect on the healthy tenocyte was evaluated by analyzing the changes in cell viability, as well as gene and protein expressions.

## 2. Results

**Figure 1:**
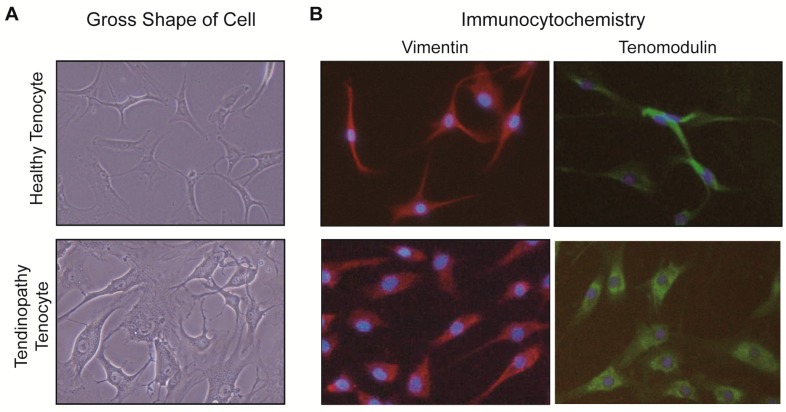
Identification of healthy and tendinopathy tenocytes. Under light microscopy, the morphology of cells from each group was observed (**A**); The morphology of the tendinopathy group had short elongated processes with more round or oval nuclei compared to the cells from healthy group (**A**,**B**); Cells from healthy and tendinopathy tenocyte cultures stained with immunocytochemical methods (**B**). The majority of cells expressed the surface marker for vimentin (red) and tenomodulin (green). The cells are counterstained with DAPI to mark the nuclei (blue). Original magnification: 400× for **A** and **B**; DAPI: 4′,6-diamidino-2-phenylindole dihydrochloride.

All tenocytes were successfully grown in culture. To confirm that the cultured cells were tenocytes, we performed immunocytochemistry (ICC). The images showed that most of the cultured cells from healthy samples and tendinopathy samples expressed the surface marker for vimentin (red) and tenomodulin (green), and had elongated appearances under microscopy (Figure 1).

### 2.1. Comparison between the Healthy Tenocyte and the Tendinopathy Tenocyte

#### 2.1.1. Gross Description of Cell Shape

We observed the cultured cells of healthy and tendinopathy samples under light microscope and fluorescence microscope when the cells reached passage 3 and when the confluency of cells was 80%. The tendinopathy cells were less spindle-shaped with round or oval nuclei, and small or less elongated processes compared with those from the healthy group (Figure 1).

#### 2.1.2. Differences of Expressions at the mRNA Level

**Figure 2:**
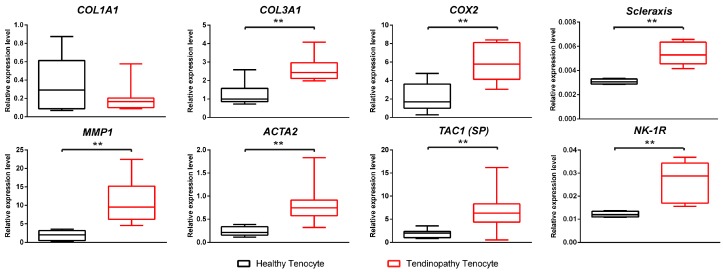
Differential gene expressions between healthy tenocyte and tendinopathy tenocyte. Relative expressions by qRT-PCR of 8 genes were analyzed using cultured tenocytes. Results show that levels of *COL3A1*, *MMP1*, *COX2*, *SCX*, *ACTA2*, *TAC1* (*SP*), and *NK-1R* were significantly increased in the cells cultured from tendinopathy tenocyte. However, *COL1A1* was decreased in the cells cultured from tendinopathy tenocyte without statistical significance. The result was expressed as the relative expression level. Data were normalized on *GAPDH* mRNA levels. Boxplot showing the median along with the first and third quartiles. Error bar means data range from minimum to maximum. ** *p* < 0.01. SP: substance P; qRT-PCR: quantitative real time-polymerase chain reaction results.

Eight genes known to be involved in the pathogenesis of tendinopathy were analyzed by quantitative real time-polymerase chain reaction (qRT-PCR). We found higher expressions of the genes including collagen type 3 α1 (*COL3A1*), prostagladin endoperoxide synthase 2 (*PTGS2* = *COX2*), scleraxis (*SCX*), matrix metalloproteinase 1 (*MMP1*), α-smooth muscle actin (*ACTA2*), tachykinin precursor 1 (*TAC1* = *SP*), and neurokinin-1 receptor (*NK-1R*) in cultured cells from the tendinopathy tendon compared to those of the cultured cells from the healthy tendon (*p* < 0.01, in all). In contrast, there was down-regulation of collagen type 1 α1 (*COL1A1*) in the tendinopathy cells compared to the healthy cells without statistical significance (*p* > 0.05). Consequently, the ratio of *COL1A1*/*COL3A1* was 13.2 times greater in the healthy cells. The relative expressions for all genes are shown in Figure 2.

### 2.2. The Effect of Substance P (SP) on the Healthy Tenocyte in the Development of Tendinopathy

#### 2.2.1. SP Increases Cellular Proliferation and Viability

**Figure 3:**
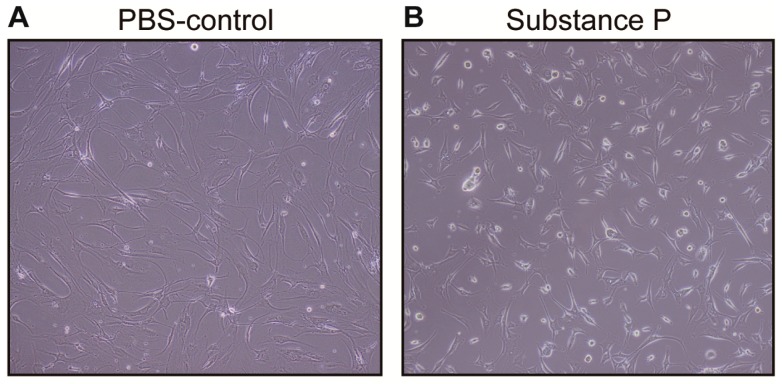
Effect of SP on the cell morphology. The healthy tenocytes were cultured in medium containing PBS-control (**A**) and SP (**B**). Morphological changes of the cultured healthy tenocyte as observed under a phase contrast microscope for 72 h. The cultured tenocytes with SP became more round or ovoid, but the cells with PBS had an elongated spindle shape. Original magnification: 100×; PBS: Phosphate buffered saline; SP: substance P.

**Figure 4:**
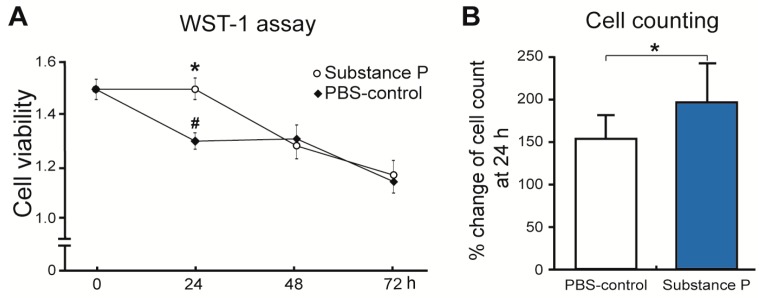
Increased cell viability and proliferation by SP in the cultured tenocytes from healthy tendon. The healthy tenocytes were cultured in medium containing SP or PBS to compare their effects on cell viability and proliferation. Then, cell viability and proliferation were monitored using Cell Counting Kit-8 assay for 72 h. At 24 h, cell viability was significantly higher in the SP group compared to the PBS-control group. However, there were no differences between the groups with and without SP at 48 h (**A**); The percent change of cell number compared to 0 h was calculated by hemocytometer 24 h after PBS or SP treatment. The result showed significantly higher percent increase of cell number in the SP-group compared to PBS-control (**B**). Each value represents the mean ± standard deviation. ^#^ *p* < 0.05 compared to 0 h, * *p* < 0.05 compared to PBS-control group at the same time point. WST-1: 2-(4-iodophenyl)-3-(4-nitrophenyl)-5-(2,4-disulfophenyl)-2*H*-tetrazolium; PBS: Phosphate buffered saline; SP: substance P.

Twenty-four hours after applying SP on the healthy tenocytes, the morphological structure of the cultured tenocytes became more round; similar to what was observed in the tendinopathy tenocytes (Figure 3). Interestingly, the viability of cell incubation with SP was significantly higher compared to the controls in a monosodium salt, 2-(4-iodophenyl)-3-(4-nitrophenyl)-5-(2,4-disulfophenyl)-2*H*-tetrazolium (WST-1) assay at 24 h (*p* < 0.01). Repeated measures analysis of variance (ANOVA) revealed that optical densities in SP treated cells increased slightly by 3.2% (*p* > 0.05), whereas the optical densities in phosphate buffered saline (PBS) treated cells decreased substantially by 12.5% at 24 h versus 0 h (*p* < 0.01) (Figure 4A). However, the intensities returned to similar levels in both SP and PBS treated groups at 48 h. Hematocytometry revealed a higher cellular proliferation rate for the SP treated group (191%) than the PBS treated group (152%) at 24 h (Figure 4B). 

#### 2.2.2. SP Up-Regulates the Relative mRNA Expressions Related to Tendinopathy Development in the Healthy Tenocyte

**Figure 5:**
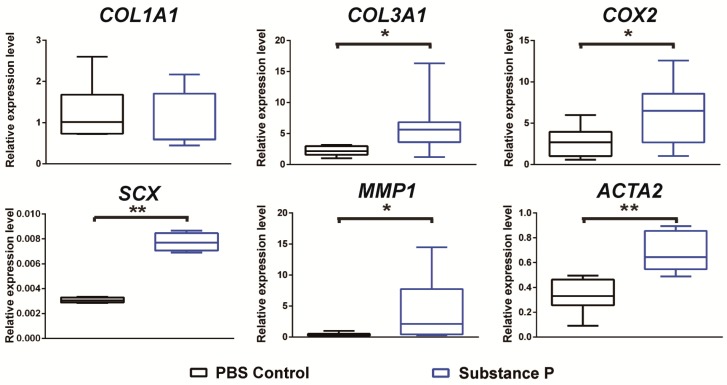
SP upregulation of genes related to tendinopathy. To determine the effects of SP on the development of tendinopathy, cultured healthy tenocytes were pretreated with or without SP 10^−7^ M for 24 h. Results show that healthy tenocytes developed similar gene expression trends as that of the tendinopathy tenocyte. Levels of *COL1A1* decreased but *COL3A1*, *COX2*, *SCX*, *MMP1*, and *ACTA2* were significantly increased as a result of treatment with SP. The result was expressed as the relative expression level after normalizing on *GAPDH* mRNA levels. Boxplot showing the median along with the first and third quartiles. Error bar means data range from minimum to maximum. * *p* < 0.05; ** *p* < 0.01; SP: substance P.

The relative expressions of six genes including *COL1A1*, *COL3A1*, *COX2*, *SCX*, *MMP1*, and *ACTA2* were measured with qRT-PCR 24 h after SP application to the culture medium of healthy tenocytes. As shown in the results comparing the healthy and tendinopathy samples, the relative mRNA expressions of *COL3A1*, *COX2*, *SCX*, *MMP1*, and *ACTA2* were increased by 1.4, 1.8, 2.7, 1.3, and 1.6 times in the SP-treated samples compared to the PBS-controls (*p* < 0.05 in all), respectively. Like the results from the earlier comparison of the healthy and tendinopathy tenocytes, the relative expression of *COL1A1* showed no statistical difference between the PBS-control and SP-treated cells (*p* > 0.05) despite the observed decrease in the SP-treated group. The *COL1A1*/*COL3A1* ratio was calculated to be 0.21 in the PBS-control cells and 0.11 in the SP-treated cells (Figure 5).

#### 2.2.3. SP Increases the Protein Levels Related to Tendinopathy Development in the Healthy Tenocyte

**Figure 6:**
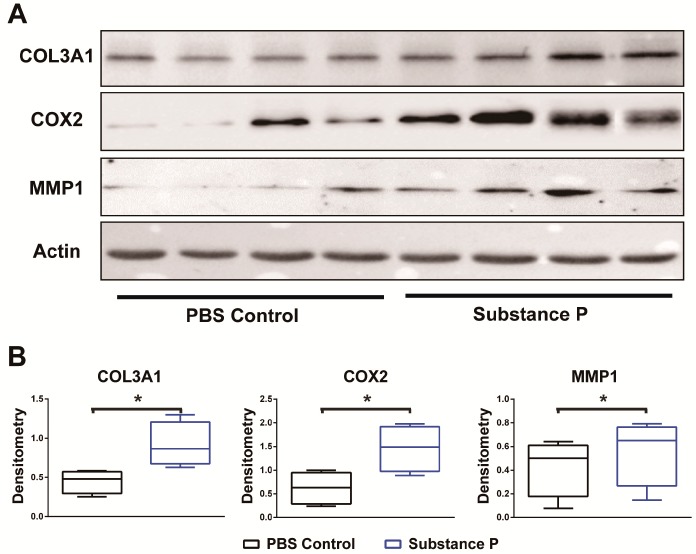
SP induced the development of tendinopathy at protein level. Western blot for the cultured healthy tenocyte with or without SP was performed with antibodies against *COL3A1*, *COX2*, and *MMP1* protein. Loading control was performed with an anti-β-actin antibody. Twenty-four hours of exposure to SP resulted in strong band densities for every protein. The results were consistent with the analysis of qRT-PCR (**A**); Densitometric analysis, as expected, revealed that *COL3A1*, *COX2*, and *MMP1* protein band intensities normalized to β-actin were increased by 203.8%, 129.3%, and 129.3% respectively in SP-treated group compared with PBS-control. Boxplot showing the median along with the first and third quartiles (**B**). Error bar means data range from minimum to maximum. * *p* < 0.05; SP: substance P.

Expression levels of tendinopathy related proteins were analyzed at 24 h after SP application on human healthy tenocytes. Of the three proteins investigated, the densitometric analyses of the band were normalized to that of the β-actin in band. Western blot analyses revealed that the intensities of *COL3A1*, *COX2*, and *MMP1* proteins were significantly greater in SP-treated cells compared to the PBS-treated cells (*p* < 0.05 in all). Details of the analyses are summarized in Figure 6.

## 3. Discussion

Using human samples, we tested our hypothesis that the neuro-mediator SP could influence the change of a normal healthy tendon into a tendinopathy tendon. For this experiment, we cultured both healthy and tendinopathy tenocytes and started by comparing the gene and protein levels. Next, we exogenously applied SP to the cells cultured from healthy tenocytes, analyzed the changes of the gene and protein expressions, and observed the effect on cell viability. Thus, in parallel with the findings in the first comparison between the healthy and tendinopathy samples, we observed similar changes in cell shape, and gene and protein expressions in the healthy tenocyte after SP application.

### 3.1. SP Is Closely Related to Tendon Healing

It was suggested that SP could stimulate healing by enhancing cellular proliferation of fibroblasts, myofibroblasts, and stem cells, as well as increase collagen formation in skin, tendon, and corneal injuries [19,20,21,22,23,24] SP also enhances angiogenesis, vasopermeability, and the synthesis of various cytokines [24,25,26,27,28]. However, although SP induces cell proliferation for tissue healing, it was also recently reported that SP is one of the causes of tendinopathy. Fong et al. applied SP to healthy tenocytes and analyzed the changes in collagen remodeling [6]. The authors indicated that SP could be initially beneficial for tendon healing, but with sustained upregulation of SP, excessive collagen remodeling may ensue and lead to the progression of tendinopathy. These results collectively reinforce the idea that SP plays a biological role in tissue injuries.

Consistent with the previous findings of SP’s role in healing, we observed higher proliferation rate of cells 24 h after SP treatment in both the WST-1 assay and hematocytometry. However, the increased proliferation capacity was highest at 24 h and disappeared at 48 h, which suggested that the sustained SP increase might be one of the reasons for hypercellularity of tendinopathy. We also assumed based on this result that the onset of action of SP was around 24 h and decided to analyze the change in gene and protein levels at 24 h. 

Another finding of this study was that the *SCX* gene was upregulated in the tendinopathy group compared to the healthy tenocyte. *SCX* is a transcription factor expressed in the progenitors and tendon tissue cells and is related to tenogenesis and collagen production. Several papers have reported that *SCX* was increased in the injured tendon as the injured tendon had increased collagen production for healing [29,30]. In this study, the tendinopathy tenocyte was fibroblastic tenocyte, which had chronic micro-injury, thus the tendinopathy tenocyte had increased *SCX*. Interestingly, SP treatment increased *SCX* expression, and further study will be warranted to elucidate their relationship.

### 3.2. SP Has a Role in the Development of Tendinopathy

Our study presents information showing that SP induces tendinopathy changes in human tenocytes at the gene and protein level. After SP treatment in our cultured healthy cells, levels of *COL3A1*, *MMP1*, and *ACTA2* genes were increased, which were also shown in the tendinopathy samples. However, the expression of *COL1A1* was decreased without statistical significance. To find whether there was post-translational modification of the *COL1A1* gene, the *COL1A1* protein level was analyzed using an enzyme-linked immunosorbent assay, and again we found a non-significant decrease of in *COL1A1* protein level after SP treatment (Supplementary Figure S1). 

*COL3A1* gene and protein increased by 1.4 and 2.2 times with each with SP treatment. Type 3 collagen is the predominant collagen in tendinopathy and produces smaller, less organized fibril in comparison to type 1 collagen. This difference in architecture between the two types of collagen has implications on the mechanical strength of the tendon [31,32].

We observed that *MMP1* was upregulated 24 h after the addition of SP. *MMP1* reflects the collagen turnover rate and has been reported to have increased activity in the degenerative suprasupinatus tendon [33]. Cury et al. [34] reported that SP contributed to the degenerative process of a tendon through its control over both the *MMP* and tissue inhibitor metalloproteinase expression levels. This could also be seen in cultured human gingival fibroblasts and could explain periodontal breakdown seen in periodontal disease. Additionally, in vitro studies of Achilles tendon and collateral ligament cells show that SP, along with CGRP, could modulate messenger RNA levels of *MMP1* and/or *MMP3* [35,36].

Another important gene found to be upregulated in tendinopathy samples, as well as in healthy samples with SP treatment, was *ACTA2*. *ACTA2* is an important cytoskeleton gene that encodes the α-smooth muscle actin [37,38,39], which is a functional marker for a fibroblast subtype that rapidly remodels the extracellular matrix [40]. The gene and the protein it encodes are found not only in smooth muscles, but also in pericytes or myofibroblasts [41]. The latter are known to play a major role in tissue healing since they are specialized cells that populate areas of persistent injury, regardless of tissue type [41,42]. Tendinopathy tendon tissues, in particular, are accompanied by increasingly conspicuous cells that mostly have a fibroblastic or myofibroblastic appearance. Therefore, an increase in *ACTA2* expression, along with increased α-smooth muscle actin production in myofibroblasts, is one of the hallmarks of tendinopathy lesions [43].

### 3.3. SP Contributed to the Development of Neurogenic Inflammation

It should be noted that SP is known to be associated with pain by triggering neurogenic inflammation [5,9,15,16,44,45,46,47,48,49]. Our results support this, as SP application increased the COX2 gene and protein level, which is the primary culprit responsible for inflammation and pain. Lui et al. reported an increase of SP and CGRP immunopositivity in rat tenocytes and the presence of immunopositive staining in the tendon fibroblasts [15]. Furthermore, the immunopositivity of SP and CGRP was found to have significant positive correlations with pain.

### 3.4. Limitations

Our experiment had several limitations. First, we only harvested the extensor carpi radialis brevis (ECRB) tendon from tennis elbow (lateral epicondylitis) surgery and used it as the tendinopathy tenocyte since the ECRB tendon of the elbow is a common tendinopathy site for surgery, along with the Achilles and rotator cuff tendons. However, it is possible that features of tenocytes from other tendinopathy sites may differ from cells harvested from the ECRB tendon. Further studies need to be conducted with a larger sample size focusing on longitudinal changes and any differences between various tendinopathy sites. Next, it is debatable that *TAC1* gene expression was considered as representative of *SP* expression in our study, given that *TAC1* not only codes for *SP*, but also encodes neurokinin A [50]. This was supported by the fact that SP is well known as the major neuropeptide synthesized from the *TAC1* gene and found several studies that regarded *TAC1* as representative of *SP* expression [51,52]. Finally, there may have been variations in the tendinopathy cells between samples as they were taken from different patients with heterogeneous characteristics, as well as different histories of steroid injections and medications. However, we assumed that all of them had significant pathognomonic changes of tendinopathy as they received surgery due to non-responsiveness to non-surgical treatment [45].

## 4. Materials and Methods

### 4.1. Experiment Overview

All subjects gave their informed consent for inclusion before they participated in the study. The study was conducted in accordance with the Declaration of Helsinki, and the protocol was approved by the institutional review board at CHA Bundang Medical Center (2013-01-174; 5 April 2013). We harvested and cultured tenocytes of healthy tendons (*n* = 10) obtained from healthy donors and tendinopathy tendons (*n* = 10) from tendinopathy patients. ICC was used to confirm that the cultured cells were tenocytes. The differences in gene expression levels related to tendinopathy development were analyzed between the cultured healthy tenocytes and tendinopathy tenocytes. To examine if SP contributed to the pathogenesis of tendinopathy, the healthy tenocytes (*n* = 4) were cultured with either PBS or SP (Sigma-Aldrich, St. Louis, MO, USA) at the concentration of 10^−7^ M, based on previous studies in Reference [6]. After PBS or SP treatment, the gene and protein expression levels between the two groups were analyzed at 24 h. These results were compared to those from the previous comparison between the healthy and tendinopathy tenocytes. The changes in cell viability between PBS-treated tenocytes and SP-treated tenocytes were also compared at 72 h.

### 4.2. Tissue Sampling

All the healthy donors and tendinopathy patients were female and had a mean age of 43.5 (±9.47) years old. They did not have any systemic or local diseases that would affect the results. Samples of healthy tendons were acquired from the tendon of the hamstrings during anterior cruciate ligament reconstruction or the palmaris longus tendon during tendon graft surgery.

**Figure 7:**
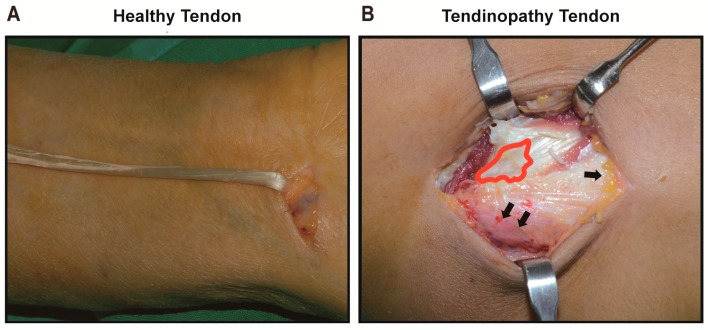
Intraoperative photos of a healthy tendon and a tendinopathy tendon. Healthy tendon was harvested during the anterior cruciate ligament reconstruction or PL tendon during tendon graft procedures and used as the healthy control (**A**); The origin of extensor carpi radialis brevis (ECRB) tendon from lateral epicondylitis patient was harvested as tendinopathy tendon. Note the white-grayish discoloration and friability with fibrosis, both of which are indicative of degeneration (red line) (**B**). Arrow indicates the subcutaneous adipose tissue and the tissue of musculotendinous junction. PL: Palmaris longus; ECRB: Extensor carpi radialis brevis.

Samples of tendinopathy tendons were harvested from patients with lateral epicondylitis during the surgical partial resection of ECRB origin by drilling the lateral condyle using the same method as the previous study in Reference [45]. Biopsy specimens were taken strictly from the ECRB tendon corresponding to structural changes observed during surgery. All harvested tendons were described as dull, gray, friable, and edematous and were different from the healthy tendons. The size of the harvested samples was around 10 × 10 × 7 mm^3^. All operations and sampling were performed by one senior surgeon (Figure 7).

Before surgical treatment, non-operative treatment was performed, including cessation of the offending activity, physiotherapy (manipulation, stretching, ultrasound, electrical stimulation, and strengthening exercises), corticosteroid injections, and anti-inflammatory agents for all patients. Corticosteroid injections were done for all patients 1–3 times, and the surgery was performed at least three months after the last injection to exclude the effect of corticosteroid injections. Patients did not receive extracorporeal shock wave therapy, platelet-rich plasma, or botulinum toxin A injections prior to surgery.

### 4.3. Cell Culture

Harvested samples were washed once in PBS, and minced into small pieces. The samples were enzymatically digested for one hour in Dulbecco’s modified Eagle’s medium (D-MEM) supplemented with 10 mg/mL Collagenase II (Gibco, Carlsbad, CA, USA) at 37 °C. Collagenase II was deactivated by adding fetal bovine serum (FBS), and the pellet was centrifuged for five minutes at 1000 rpm. The cells obtained were seeded into a 100 φ dish and cultured with D-MEM supplemented with 10% FBS, 50 mM ascorbic acid-2-phosphate (Gibco), and 1% Antibiotic-Antimycotic solution (Gibco) for one week. The culture medium was changed every three days until confluence. Both healthy and tendinopathy cell samples were cultured until passage 3 and used for experimentation.

### 4.4. Cell Morphology

Daily examination of the cell morphology was conducted under an inverted phase-contrast light microscope for all samples (Nikon Eclipse TE300; Nikon, Tokyo, Japan).

### 4.5. Immunocytochemistry (ICC)

During cell culture, we confirmed the identity of tenocytes using the ICC with 2 × 10^5^ cells. After transferring 200 μL of cell culture to the 8-wells of a chamber slide, the cells were grown to confluence with the addition of fresh media for one day. After washing with PBS, the cells were fixed with 4% formaldehyde. Permeabilization of the membranes was done by incubating the slides with 0.25–0.5% Triton X-100 in PBS for 10 min, followed by blocking with rabbit normal serum, donkey normal serum, for vimentin and tenomodulin, respectively. The cells were incubated in the dark with mouse anti-vimentin monoclonal antibody (1:100 dilution; Dako, Glostrup, Denmark; code: M0725) and goat anti-tenomodulin antibody (1:25 dilution; Santa Cruz Biotechnology, Santa Cruz, CA, USA; code: sc-49325) for 60 min at 37 °C. After additional washing, the secondary antibodies were incubated in the dark for 30 min at 37 °C, and mounted in a Vectashield H-1000 mounting medium for fluorescence (Vector Laboratories, Burlingame, CA, USA). Analysis was done using an Olympus CKX41 microscope equipped with epifluorescence and an Olympus XC10 digital camera. To determine the expression of vimentin or tenomodulin in the cultured cells, we randomly sampled 40 views of cell staining from two groups (healthy tenocyte, tendinopathy tenocyte), with approximately 1000 cells each.

### 4.6. Cell Counting Kit-8 (CCK-8) Assay

Cell viabilities were measured using a WST-1 assay under a serum starved condition. Healthy tenocytes were seeded into 96-well plates at a density of 5 × 10^3^ cells per well and treated with SP for 0 (baseline), 24, 48, and 72 h. After incubation, 10 μL of the WST-1 solution (Dojindo Molecular Technologies, Rockville, MD, USA) was added to each well, and the plates were incubated for 3 h at 37 °C in 5% CO_2_. The optical densities were measured at 450 nm using an EON Gen 5.2 software (Bio Tek, Vermont, VT, USA). Readings were taken at least three times at all time points for each sample.

### 4.7. Cell Counting

Cell count was determined by the percentage change in cell number at 24 h from the cell number at 0 h. The D-MEM media in the 100 φ dish was removed and cells were detached by adding trypsin-ethylenediaminetetraacetic acid (EDTA) at 37 °C in 5% CO_2_ at 95% relative humidity for 5 min. Cell counting was performed using a hematocytometry under starved conditions.

### 4.8. RNA Isolation and qRT-PCR 

Total mRNA was isolated from the cultured tenocytes and homogenized in TRIzol reagent (Invitrogen, Carlsbad, CA, USA) as per the manufacturer’s instructions. A total of 1 μg RNA from each sample was reverse-transcribed into complementary DNA (cDNA) using an iScript™ cDNA Synthesis Kit (Bio-Rad Applied Science, Mannheim, Germany). The PCR was carried out in triplicate using the Bio-Rad CFX96 Real-Time PCR Detection System. Amplification of total RNA was performed using the Taq Man Gene Expression Assay (Applied Biosystems, Warrington, UK) for the analyses of glyceraldehyde-3-phosphate dehydrogenase (*GAPDH*) (ABI code: Hs99999905_m1), *COL1A1* (Hs00164004_m1), *COL3A1* (Hs00943809_m1), *PTGS2* (*COX2*) (Hs00153133_m1), *SCX* (Hs03054634_g1), *MMP1* (Hs00899658_m1), *ACTA2* (Hs00426835_g1), *TAC1* (*SP*) (Hs00153133_m1), and *NK-1R* (Hs00185530_m1), respectively. Transcript levels were normalized against *GAPDH* expression, which was expressed stably across the sample, and gene expression was calculated using 2^−ΔΔ*C*t^. For more accurate analysis, transcript levels were measured at least three times for each sample.

### 4.9. Western Blot

Cells were lysed by protein extraction buffer (Pro-Prep, iNtRON Biotechnology, Gyeonggi-do, Korea). After centrifugation at 4 °C and 13,000 rpm for 15 min, the protein content of lysed cells was determined by the Bradford assay. Same amounts of total protein were run on 10% SDS polyacrylamide gels and transferred to a nitrocellulose membrane. The membranes were blocked with 5% non-fat milk powder in room temperature, and then incubated with primary antibodies in tris-buffered saline-tween 20 (TBS-T) overnight at 4 °C. We used the following primary antibodies: *COL3A1* protein 1:1000 (Gene Tex, Irvine, CA, USA), *COX2* protein 1:1000 (Gene Tex), and *MMP1* protein 1:1000 (Gene Tex).

The membranes were then washed in TBS-T and incubated with 1:5000 goat anti-rabbit IgG and 1:5000 rabbit anti-mouse IgG (Santa Cruz Biotechnology) secondary antibodies for 1 h at room temperature. Next, the bands of membranes were visualized by an ECL (enhanced luminol-based chemiluminescence) detection kit (Bio-Rad Laboratories, Hercules, CA, USA). The quantification of protein was conducted by densitometric digital analysis of protein bands using the ChemiDoc™ XRS+ with Image Lab™ Software ver. 6.0 (Bio-Rad Laboratories). Equal loading was confirmed by re-probing the membrane with β-actin (Cell Signaling technology, Danvers, MA, USA). Western blot analysis was performed at least three times for each sample, and densitometric measurements were taken for each three different bands.

### 4.10. Statistical Analysis

Data were collected and analyzed using R software (version 2.11.1; R Foundation for Statistical Computing, Vienna, Austria). The Mann–Whitney *U* test was used to compare the healthy and tendinopathy samples, and a Wilcoxon signed rank test was used to evaluate the effect of SP on the healthy tenocytes. To analyze the time-dependent effects of SP on cell proliferation, repeated measures ANOVA was used to determine whether the results were significantly different at the four time points and the Wilcoxon signed rank test was used for the hemocytometry. Differences were considered statistically significant when the *p* value was less than 0.05 (*p* < 0.05).

## 5. Conclusions

In summary, our results showed that SP enhanced cellular proliferation and increased remodeling via the upregulation of *MMP1* to repair damaged tissues. These enhanced proliferative and remodeling effects may be beneficial to acute tendon injury repair. However, in repetitive tendon injuries such as tendinopathy, SP had an eventual detrimental effect on tendon healing due to the increase of Type 3 collagen, which is related to the tendon structural weakness instead of Type 1 collagen. In addition, SP was responsible for the symptoms of tendinopathy with the upregulation of *COX2*. In conclusion, our results suggest that an abnormal increase in SP expression plays a crucial role in the development of tendinopathy and associated symptoms, suggesting its use as a possible future therapeutic target for potential treatments.

## Supplementary Materials

Supplementary materials can be found at www.mdpi.com/1422-0067/18/6/1241/s1.

## References

[1] Maffulli N., Wong J., Almekinders L.C. (2003). Types and epidemiology of tendinopathy. Clin. Sports Med..

[2] Lysholm J., Wiklander J. (1987). Injuries in runners. Am. J. Sports Med..

[3] Kannus P., Jozsa L. (1991). Histopathological changes preceding spontaneous rupture of a tendon. A controlled study of 891 patients. J. Bone Jt. Surg. Am..

[4] Kvist M., Jozsa L., Jarvinen M.J., Kvist H. (1987). Chronic Achilles paratenonitis in athletes: A histological and histochemical study. Pathology.

[5] Uchio Y., Ochi M., Ryoke K., Sakai Y., Ito Y., Kuwata S. (2002). Expression of neuropeptides and cytokines at the extensor carpi radialis brevis muscle origin. J. Shoulder Elbow Surg..

[6] Fong G., Backman L.J., Hart D.A., Danielson P., McCormack B., Scott A. (2013). Substance P enhances collagen remodeling and MMP-3 expression by human tenocytes. J. Orthop. Res..

[7] Backman L.J., Fong G., Andersson G., Scott A., Danielson P. (2011). Substance P is a mechanoresponsive, autocrine regulator of human tenocyte proliferation. PLoS ONE.

[8] Ackermann P.W. (2013). Neuronal regulation of tendon homoeostasis. Int. J. Exp. Pathol..

[9] Lian O., Dahl J., Ackermann P.W., Frihagen F., Engebretsen L., Bahr R. (2006). Pronociceptive and antinociceptive neuromediators in patellar tendinopathy. Am. J. Sports Med..

[10] Sanchis-Alfonso V., Rosello-Sastre E., Subias-Lopez A. (2001). Neuroanatomic basis for pain in patellar tendinosis (“jumper’s knee”): A neuroimmunohistochemical study. Am. J. Knee Surg..

[11] Schubert T.E., Weidler C., Lerch K., Hofstadter F., Straub R.H. (2005). Achilles tendinosis is associated with sprouting of substance P positive nerve fibres. Ann. Rheum. Dis..

[12] Alpantaki K., McLaughlin D., Karagogeos D., Hadjipavlou A., Kontakis G. (2005). Sympathetic and sensory neural elements in the tendon of the long head of the biceps. J. Bone Jt. Surg. Am..

[13] Fedorczyk J.M., Barr A.E., Rani S., Gao H.G., Amin M., Amin S., Litvin J., Barbe M.F. (2010). Exposure-dependent increases in IL-1β, substance P, CTGF, and tendinosis in flexor digitorum tendons with upper extremity repetitive strain injury. J. Orthop. Res..

[14] Singaraju V.M., Kang R.W., Yanke A.B., McNickle A.G., Lewis P.B., Wang V.M., Williams J.M., Chubinskaya S., Romeo A.A., Cole B.J. (2008). Biceps tendinitis in chronic rotator cuff tears: A histologic perspective. J. Shoulder Elbow Surg..

[15] Lui P.P., Chan L.S., Fu S.C., Chan K.M. (2010). Expression of sensory neuropeptides in tendon is associated with failed healing and activity-related tendon pain in collagenase-induced tendon injury. Am. J. Sports Med..

[16] Ackermann P.W., Li J., Lundeberg T., Kreicbergs A. (2003). Neuronal plasticity in relation to nociception and healing of rat Achilles tendon. J. Orthop. Res..

[17] Burssens P., Steyaert A., Forsyth R., van Ovost E.J., Depaepe Y., Verdonk R. (2005). Exogenously administered substance P and neutral endopeptidase inhibitors stimulate fibroblast proliferation, angiogenesis and collagen organization during Achilles tendon healing. Foot Ankle Int..

[18] Bring D.K., Paulson K., Renstrom P., Salo P., Hart D.A., Ackermann P.W. (2012). Residual substance P levels after capsaicin treatment correlate with tendon repair. Wound Repair Regen..

[19] Carlsson O., Schizas N., Li J., Ackermann P.W. (2011). Substance P injections enhance tissue proliferation and regulate sensory nerve ingrowth in rat tendon repair. Scand. J. Med. Sci. Sports.

[20] Hong H.S., Lee J., Lee E., Kwon Y.S., Lee E., Ahn W., Jiang M.H., Kim J.C., Son Y. (2009). A new role of substance P as an injury-inducible messenger for mobilization of CD29^+^ stromal-like cells. Nat. Med..

[21] Khalil Z., Helme R. (1996). Sensory peptides as neuromodulators of wound healing in aged rats. J. Gerontol. A Biol. Sci. Med. Sci..

[22] Nilsson J., von Euler A.M., Dalsgaard C.J. (1985). Stimulation of connective tissue cell growth by substance P and substance K. Nature.

[23] Steyaert A.E., Burssens P.J., Vercruysse C.W., Vanderstraeten G.G., Verbeeck R.M. (2006). The effects of substance P on the biomechanic properties of ruptured rat Achilles’ tendon. Arch. Phys. Med. Rehabil..

[24] Ziche M., Morbidelli L., Pacini M., Geppetti P., Alessandri G., Maggi C.A. (1990). Substance P stimulates neovascularization in vivo and proliferation of cultured endothelial cells. Microvasc. Res..

[25] Lotz M., Vaughan J.H., Carson D.A. (1988). Effect of neuropeptides on production of inflammatory cytokines by human monocytes. Science.

[26] Lai X.N., Wang Z.G., Zhu J.M., Wang L.L. (2003). Effect of substance P on gene expression of transforming growth factor β-1 and its receptors in rat’s fibroblasts. Chin. J. Traumatol..

[27] Cuesta M.C., Quintero L., Pons H., Suarez-Roca H. (2002). Substance P and calcitonin gene-related peptide increase IL-1β, IL-6 and TNFα secretion from human peripheral blood mononuclear cells. Neurochem. Int..

[28] Saria A., Lundberg J.M., Skofitsch G., Lembeck F. (1983). Vascular protein linkage in various tissue induced by substance P, capsaicin, bradykinin, serotonin, histamine and by antigen challenge. Naunyn Schmiedebergs Arch. Pharmacol..

[29] Ahmed A.S., Li J., Abdul A.M., Ahmed M., Ostenson C.G., Salo P.T., Hewitt C., Hart D.A., Ackermann P.W. (2017). Compromised neurotrophic and angiogenic regenerative capability during tendon healing in a rat model of type-II diabetes. PLoS ONE.

[30] Loiselle A.E., Bragdon G.A., Jacobson J.A., Hasslund S., Cortes Z.E., Schwarz E.M., Mitten D.J., Awad H.A., O’Keefe R.J. (2009). Remodeling of murine intrasynovial tendon adhesions following injury: MMP and neotendon gene expression. J. Orthop. Res..

[31] Lui P.P., Chan L.S., Lee Y.W., Fu S.C., Chan K.M. (2010). Sustained expression of proteoglycans and collagen type III/type I ratio in a calcified tendinopathy model. Rheumatol..

[32] Riley G.P., Harrall R.L., Constant C.R., Chard M.D., Cawston T.E., Hazleman B.L. (1994). Tendon degeneration and chronic shoulder pain: Changes in the collagen composition of the human rotator cuff tendons in rotator cuff tendinitis. Ann. Rheum. Dis..

[33] Riley G.P., Curry V., DeGroot J., van El B., Verzijl N., Hazleman B.L., Bank R.A. (2002). Matrix metalloproteinase activities and their relationship with collagen remodelling in tendon pathology. Matrix Biol..

[34] Cury P.R., Canavez F., de Araujo V.C., Furuse C., de Araujo N.S. (2008). Substance P regulates the expression of matrix metalloproteinases and tissue inhibitors of metalloproteinase in cultured human gingival fibroblasts. J. Periodontal Res..

[35] Hart D.A., Kydd A., Reno C. (1999). Gender and pregnancy affect neuropeptide responses of the rabbit Achilles tendon. Clin. Orthop. Relat. Res..

[36] Hart D.A., Reno C. (1998). Pregnancy alters the in vitro responsiveness of the rabbit medial collateral ligament to neuropeptides: Effect on mRNA levels for growth factors, cytokines, iNOS, COX-2, metalloproteinases and TIMPs. Biochim. Biophys. Acta.

[37] Gabbiani G., Ryan G.B., Majne G. (1971). Presence of modified fibroblasts in granulation tissue and their possible role in wound contraction. Experientia.

[38] Grinnell F. (1994). Fibroblasts, myofibroblasts, and wound contraction. J. Cell Biol..

[39] Schurch W., Seemayer T.A., Gabbiani G. (1998). The myofibroblast: A quarter century after its discovery. Am. J. Surg. Pathol..

[40] Arora P.D., McCulloch C.A. (1994). Dependence of collagen remodelling on α-smooth muscle actin expression by fibroblasts. J. Cell. Physiol..

[41] Tomasek J.J., Gabbiani G., Hinz B., Chaponnier C., Brown R.A. (2002). Myofibroblasts and mechano-regulation of connective tissue remodelling. Nat. Rev. Mol. Cell Biol..

[42] Gabbiani G. (1981). The myofibroblast: A key cell for wound healing and fibrocontractive diseases. Prog. Clin. Biol. Res..

[43] Khan K.M., Cook J.L., Bonar F., Harcourt P., Astrom M. (1999). Histopathology of common tendinopathies. Update and implications for clinical management. Sports Med..

[44] Gotoh M., Hamada K., Yamakawa H., Inoue A., Fukuda H. (1998). Increased substance P in subacromial bursa and shoulder pain in rotator cuff diseases. J. Orthop. Res..

[45] Han S.H., An H.J., Song J.Y., Shin D.E., Kwon Y.D., Shim J.S., Lee S.C. (2012). Effects of corticosteroid on the expressions of neuropeptide and cytokine mRNA and on tenocyte viability in lateral epicondylitis. J. Inflamm..

[46] Ljung B.O., Alfredson H., Forsgren S. (2004). Neurokinin 1-receptors and sensory neuropeptides in tendon insertions at the medial and lateral epicondyles of the humerus. Studies on tennis elbow and medial epicondylalgia. J. Orthop. Res..

[47] Fearon A.M., Twin J., Dahlstrom J.E., Cook J.L., Cormick W., Smith P.N., Scott A. (2014). Increased substance P expression in the trochanteric bursa of patients with greater trochanteric pain syndrome. Rheumatol. Int..

[48] Singh J.A., Noorbaloochi S., Knutson K.L. (2017). Cytokine and neuropeptide levels are associated with pain relief in patients with chronically painful total knee arthroplasty: A pilot study. BMC Musculoskelet. Disord..

[49] Warner S.C., Walsh D.A., Laslett L.L., Maciewicz R.A., Soni A., Hart D.J., Zhang W., Muir K.R., Dennison E.M., Leaverton P. (2017). Pain in knee osteoarthritis is associated with variation in the neurokinin 1/substance P receptor (TACR1) gene. Eur. J. Pain.

[50] Gonzalez-Santana A., Marrero-Hernandez S., Dorta I., Hernandez M., Pinto F.M., Baez D., Bello A.R., Candenas L., Almeida T.A. (2016). Altered expression of the tachykinins substance P/neurokinin A/hemokinin-1 and their preferred neurokinin 1/neurokinin 2 receptors in uterine leiomyomata. Fertil. Steril..

[51] Mousavizadeh R., Backman L., McCormack R.G., Scott A. (2015). Dexamethasone decreases substance P expression in human tendon cells: An in vitro study. Rheumatol..

[52] Wan Y., Meng F., Wu N., Zhou T., Venter J., Francis H., Kennedy L., Glaser T., Bernuzzi F., Invernizzi P. (2017). Substance P increases liver fibrosis by differential changes in senescence of cholangiocytes and hepatic stellate cells. Hepatology.

