# Supplementary figures and images for: The Implication of Substance P in the Development of Tendinopathy: A Case Control Study
Source: Int J Mol Sci. 2017 Jun 9;18(6):1241. doi: 10.3390/ijms18061241 (PMC5486064; doi:10.3390/ijms18061241)

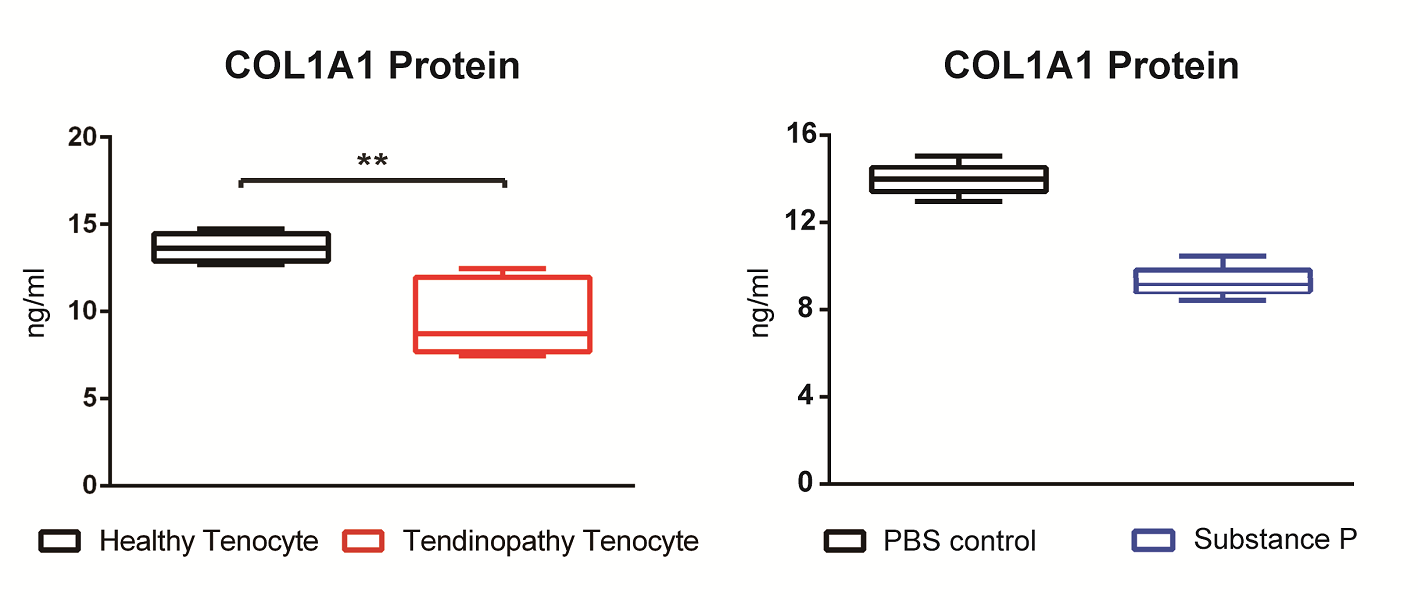

Supplement: Supplementary file 1 [file ijms-18-01241-s001.zip › Supplementary Figure S1.tif]
